# Review of Mendelian Randomization Studies on Ovarian Cancer

**DOI:** 10.3389/fonc.2021.681396

**Published:** 2021-08-11

**Authors:** Jian-Zeng Guo, Qian Xiao, Song Gao, Xiu-Qin Li, Qi-Jun Wu, Ting-Ting Gong

**Affiliations:** ^1^Department of Clinical Epidemiology, Shengjing Hospital of China Medical University, Shenyang, China; ^2^College of Life and Health Sciences, Northeastern University, Shenyang, China; ^3^Clinical Research Center, Shengjing Hospital of China Medical University, Shenyang, China; ^4^Department of Obstetrics and Gynecology, Shengjing Hospital of China Medical University, Shenyang, China

**Keywords:** causality, instrumental variables, mendelian randomization, ovarian cancer, risk factors, single-nucleotide polymorphisms

## Abstract

Ovarian cancer (OC) is one of the deadliest gynecological cancers worldwide. Previous observational epidemiological studies have revealed associations between modifiable environmental risk factors and OC risk. However, these studies are prone to confounding, measurement error, and reverse causation, undermining robust causal inference. Mendelian randomization (MR) analysis has been established as a reliable method to investigate the causal relationship between risk factors and diseases using genetic variants to proxy modifiable exposures. Over recent years, MR analysis in OC research has received extensive attention, providing valuable insights into the etiology of OC as well as holding promise for identifying potential therapeutic interventions. This review provides a comprehensive overview of the key principles and assumptions of MR analysis. Published MR studies focusing on the causality between different risk factors and OC risk are summarized, along with comprehensive analysis of the method and its future applications. The results of MR studies on OC showed that higher BMI and height, earlier age at menarche, endometriosis, schizophrenia, and higher circulating β-carotene and circulating zinc levels are associated with an increased risk of OC. In contrast, polycystic ovary syndrome; vitiligo; higher circulating vitamin D, magnesium, and testosterone levels; and HMG-CoA reductase inhibition are associated with a reduced risk of OC. MR analysis presents a2 valuable approach to understanding the causality between different risk factors and OC after full consideration of its inherent assumptions and limitations.

## Introduction

Ovarian cancer (OC), the eighth most common type and eighth leading cause of cancer-related mortality in women, is considered the deadliest gynecological cancer. Three main types of OC have been identified, specifically epithelial, germ cell, and sex cord-stromal, with epithelial tumors comprising about 95% of OC cases (1). Epithelial OC is classified into four primary histological subtypes: serous, endometrioid, mucinous, and clear cell carcinoma (1). Serous tumors can be categorized into high-grade serous carcinomas (HGSC) and low-grade serous carcinomas (LGSC) (1, 2), with HGSCs accounting for 70%–80% of all subtypes of epithelial OC and LGSCs for less than 5% cases. Endometrioid, mucinous, and clear cell subtypes account for 10%, 3%, and 10% cases, respectively (2). According to Global Cancer Statistics 2020, the estimated number of new OC cases in 2020 is 313,959, accounting for 3.4% of all new female cancer cases, and the OC death toll in 2018 is estimated as 184,799, representing 4.7% of all female cancer deaths (3). The symptoms of this disease are usually indistinct and diagnosed at the late stages, having spread at the time of clinical diagnosis in 75% of cases (1). The survival rate of patients with OC is related to stage at diagnosis. For instance, in the United States, the 5-year survival rate of a small proportion of women with stage I OC exceeds 90%. The 5-year survival rate of patients with regional disease is 75%–80% while that of patients with distant metastasis is only 25%. Although the prognosis of early OC is good, overall 5-year survival rate is only 48.6%, highlighting the critical need to develop effective prevention strategies to reduce the public health burden of OC.

OC is a multifactorial disorder influenced by both genetic predisposition and modifiable exposures. Identification of causative risk factors amenable to modification is thus essential for prevention of this disease. Randomized controlled trials (RCTs) can be uniformly applied to determine whether certain exposures are causal factors for diseases of public health interest. While RCTs remain the gold standard research design for inferring causality, they are extremely expensive, time-consuming, and associated with a high failure rate (>50% due to lack of efficacy) (4, 5). In addition, RCTs often involve multi-effect interventions (such as drugs that modify multiple biomarkers), which may challenge the causal inferences of any single biomarker. Finally, RCTs are not always feasible or ethical (6, 7). Observational studies provide another opportunity to clarify the relationship between exposure and disease (8). These studies provide a wealth of information on associations between disease exposure and outcome but cannot be interpreted as indicating causality owing to limitations introduced by confounding and reverse causality (9, 10).

To overcome the limitations of observational design, genetic variants have been proposed as potential instrumental variables (IV), usually single-nucleotide polymorphisms (SNPs), to simulate the effects of modifiable environmental exposures on disease susceptibility, referred to as Mendelian randomization (MR) (11). MR offers a number of advantages over observational epidemiology. First, although reverse causality cannot be completely avoided, MR can still avoid the bias caused by reverse causality to a certain extent (12). Second, MR studies are relatively immune to common behavioral, physiological, and socioeconomic confounders owing to random assignment of alleles at meiosis. Third, in most cases, genetic variants are precisely measured and reported and thus not subject to bias and errors, which is especially useful in evaluating risk factors of long-term effects (13). Therefore, MR design resembling RCT can aid in strengthening causal inferences on the roles of modifiable exposures (14), not only with significantly reduced concerns in terms of ethical, applicability, and financial issues but also for examination of causal factors for phenotypes that are not appropriate for RCTs, such as height.

MR uses germline genetic variants as instruments (i.e., proxies) for exposures (e.g., environmental factors, biological traits, or drug pathways) to examine the causal effects of these exposures on health outcomes (disease incidence or progression) (15). Exposure is determined as causal if its association with outcomes is statistically significant and can be explained entirely by the two associations of genetic variants: (1) exposure and (2) outcome (16, 17). The MR technique relies on a number of assumptions for accuracy. The rationale underlying MR and required IV assumptions are as follows:

I. IVs (SNPs being used) should be clearly and quantifiably linked to the exposure(s) in question.

II. IVs should not be linked in any way to confounding variables.

III. IVs should be linked to outcomes only through the exposure(s) in question.

To estimate a causal effect with IV analysis, additional assumptions are required. One such assumption is that:

IV. The associations are linear and not affected by statistical interactions (6).

**Figure 1:**
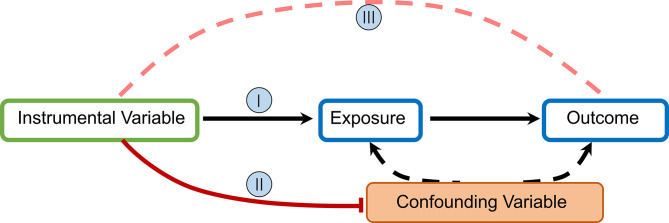
Directed acyclic graph depicting MR principles and underlying IV assumptions (I–III).

In MR studies, researchers initially identify and extract information for SNPs associated with exposure at the genome-wide significance level (*p* = 5×10^−8^) and subsequently evaluate the relationship between these SNPs and outcomes to obtain odds ratios (OR) and mean differences (**Figure 1**).

## Application of MR in OC

Although epidemiological research has revealed a wealth of biomarkers associated with increased or decreased risk of OC, causality remains largely undefined. Over the past few decades, genome-wide association studies (GWAS) have made an important contribution to the identification of genetic variants associated with numerous potential risk factors for health-related outcomes. GWAS results have facilitated the application of MR in evaluating causal relationships between modifiable exposures and outcomes. During recent years, numerous MR studies focusing on OC have been conducted (18). In addition, development of new methodologies in MR research has challenged the previously reported causality of certain biomarkers. Therefore, it is essential to record research progress and focus on the quality and effectiveness of MR. In this review, we have sorted and analyzed evidence from MR research on OC published in the literature, focused on its advantages and limitations, and designed strict literature retrieval strategies and selection criteria.

### Search Strategy and Selection Criteria

Original studies were identified by searching for relevant articles up to February 2, 2021, in the PubMed database. The search algorithms for PubMed database were as follows: “Mendelian randomization” or “genetic instrumental variable” or a related term (e.g., “genetic instrument”) and “Ovarian Cancer” or “Ovarian Neoplasm” or “Ovary Cancer” or “Ovary Neoplasm” or “Cancer, Ovary” or “Neoplasm, Ovary”, with no restriction on subheadings. All retrieved articles were checked for relevant citations and studies not included in the above electronic sources were searched manually. We included studies based on the following criteria: (1) those using MR methodology and instrumental variable analysis to evaluate risk factors of OC and (2) those performed on the basis of observational study design. The search strategy and selection criteria have been checked by two independent authors and, if necessary, the inconsistent part would be judged by third authors. A total of 30 articles were finally included and classified according to type of exposure (**Table 1**).

**Table 1:** Characteristics of Mendelian randomization studies included in the review.

Author [ref], year	Exposure and unit	Outcome	Sample size for the outcome data		Sources	SNPs	Estimate (95%CI)	MR methods
			Cases	Control				
Zhang et al. (19), 2015	Telomere length	Overall OC	4269	9123	FOCI	11	1.13 (0.87 to 1.47)	IVW
		Clear-cell OC	356				1.65 (0.78 to 3.51)	
		Endometrioid OC	715				1.30 (0.75 to 2.24)	
		Serous cancer OC	2556				1.19 (0.86 to 1.65)	
Ong et al. (20), 2016	Vitamin D (20-nmol/L decrease in 25(OH)D)	Overall OC	10065	21654	OCAC	10	1.27 (1.06 to 1.51)	IVW/WTR
		HGSC	4121				1.54 (1.19 to 2.01)	
		Mucinous OC	662				1.00 (0.70 to1.43)	
		Clear-cell OC	621				1.27 (0.72 to 2.24)	
		Endometrioid OC	1350				1.20 (0.81 to1.78)	
		Serous OC	5828				1.21 (0.84 to 1.76)	
		Others OC	1604				1.10 (0.76 to 1.60)	
Gao et al. (21), 2016	Adult BMI	Overall OC	4369	9123	FOCI	77	1.35 (1.05 to 1.72)	IVW
		Clear-cell OC	356				1.68 (0.84 to 3.36)	
		Endometrioid OC	715				1.34 (0.80 to 2.26)	
		Serous OC	2556				1.30 (0.97 to 1.76)	
	Childhood BMI	OC	4369	9123		15	1.07 (0.82 to 1.39)	
	Birthweight					7	1.07 (0.69 to 1.65)	
	Waist-hip ratio					14	1.19 (0.73 to 1.94)	
Dixon et al. (22), 2016	BMI (per 5 units BMI)	Non-HGSC	14047	23003	OCAC	87	1.29 (1.03 to 1.61)	
		Low-grade/borderline serous OC					1.93 (1.33 to 2.81)	
Dimitrakopoulou et al. (23), 2017	Vitamin D	Overall OC	4369	9123	FOCI	4	1.12 (0.86 to 1.47)	IVW/ Likelihood
		Clear-cell OC					0.99 (0.46 to 2.11)	
		Endometrioid OC					0.83 (0.48 to 1.43)	
		Serous OC					1.26 (0.91 to 1.76)	
Day et al. (24), 2017	Age at menarche adjusted for genetically predicted BMI	Overall OC	18175	26134	OCAC	389	0.93 (0.88 to 0.98)	
		Serous OC					0.92 (0.86 to 0.98)	
		Endometrial OC					0.78 (0.70 to 0.87)	
Haycock et al. (25), 2017	Telomere Length	Serous LMP OC	972	30816	OCAC	16	4.35 (2.39 to 7.94)	IVW
Ong et al. (26), 2018	Coffee consumption (an additional cup of coffee per day)	Overall OC	20683	23379	OCAC	4	0.92 (0.79 to 1.06)	WTR
		HGSC	7488			4	0.90 (0.73 to 1.10)	
	Coffeine consumption (an additional 80mg caffeine)	Overall OC	20683			2	1.01 (0.92 to 1.11)	
		HGSC	7488			2	0.99 (0.86 to 1.13)	
Ong et al. (27), 2018	Circulating 25-hydroxyvitamin D	Overall OC	1031	264638	UK Biobank	5	1.10 (0.80 to 1.51)	IVW/ WTR
Dixon-Suen et al. (28), 2018	Height (per 5 cm increase in height)	Overall OC	16395	23003	OCAC	609	1.06 (1.01 to 1.11)	IVW
		Invasive OC	14549	23003			1.06 (1.01 to 1.11)	
		Borderline OC	1680	16463			1.15 (1.02 to 1.29)	
		Clear cell OC	948	22051			1.20 (1.04 to 1.38)	
		Low-grade/borderline serous OC	1408	21131			1.15 (1.01 to 1.30)	
		Invasive/borderline mucinous OC	1567	22410			1.08 (0.96 to 1.21)	
		HGSC	7933	23003			1.05 (0.99 to 1.11)	
		Invasive endometrioid OC	2059	23003			1.05 (0.95 to 1.16)	
Yarmolinsky et al. (29), 2019	Genetic liability to endometriosis	Invasive OC	22406	40941	OCAC	10	1.10 (1.06 to 1.15)	IVW
		Clear-cell OC					1.49 (1.29 to 1.73)	
		Endometrioid OC					1.14 (1.04 to 1.24)	
		LMP OC					1.12 (1.04 to 1.22)	
		HGSC					1.07 (1.02 to 1.12)	
	Lifetime smoking exposure	Invasive OC				115	1.36 (1.04 to 1.78)	
		HGSC					1.44 (1.05 to 1.98)	
	Earlier age at menarche	Endometrioid OC				329	1.19 (1.05 to 1.36)	
	Later age at natural menopause	Endometrioid OC				35	1.09 (1.02 to 1.16)	
	BMI	Invasive OC				66	1.23 (1.07 to 1.42)	
		HGSC					1.26 (1.06 to 1.50)	
		Endometrioid OC					1.48 (1.07 to 2.06)	
		LMP OC					1.39 (1.04 to 1.85)	
	Height	Clear-cell OC				345	1.36 (1.15 to 1.61)	
	PCOS	Endometrioid OC				11	0.89 (0.82 to 0.96)	
		LGSC					1.13 (1.00 to 1.25)	
	C-reactive protein	Overall OC				8	0.97 (0.93 to 1.02)	
		Endometrioid OC					0.90 (0.82 to 1.00)	
	Sex hormone binding globulin	Overall OC				8	1.04 (0.88 to 1.35)	
	Circulating 25-hydroxyvitamin D	Overall OC				5	1.02 (0.72 to 1.44)	
	Type 2 diabetes	Overall OC				10	1.00 ( 0.97 to 1.02)	
	Parity	Overall OC				2	0.66 (0.26 to 1.69)	
Harris et al. (30), 2019	PCOS	Invasive OC	22406	40941	OCAC	14	0.92 (0.85 to 0.99)	IVW
		HGSC					0.91 (0.82 to 0.998)	
		Endometrioid OC					0.77 (0.65 to 0.92)	
Neuhausen et al. (31), 2019	Schizophrenia	Overall OC	25509	40941	OCAC/CIMBA	66	1.09 (1.04 to 1.14)	IVW
Qian et al. (32), 2019	Observed BMI	OC (premenopausal *BRCA1/2* mutation carriers)	2923 *BRCA1*:2319 *BRCA2*: 604	19649 *BRCA1*:12357 *BRCA2*: 7308	CIMBA/UK Biobank	93	1.25 (1.06 to 1.48)	IVW
	Genetically predicted BMI						1.59 (1.08 to 2.33)	
Ong et al. (33), 2019	Coffee	Overall OC	1031	141351	UK Biobank	6	0.82 (0.67 to 1.00)	IVW/WTR
Yang et al. (34), 2019	Age at menarche	Overall OC	1044	1172	Chinese GWAS	25	0.81 (0.67 to 0.97)	IVW
			29396	68502	OCAC	390	0.94 (0.90 to 0.98)	
Wen et al. (35), 2020	Vitiligo	Overall OC	25509	40901	OCAC	32	0.95 (0.93 to 0.97)	IVW/WTR
Yarmolinsky et al. (36), 2020	Genetically Proxied Inhibition of HMG-CoA Reductase	Epithelial OC	22406 3887	40941 27561	OCAC CIMBA	95	0.60 (0.43 to 0.83)	IVW
		OC (*BRCA1/2* mutation carriers)					0.69 (0.51 to 0.93)	
Guo et al. (37), 2020	Iron	Invasive OC	25509	40941	OCAC	3	0.99 (0.90 to 1.09)	IVW
		LMP OC					1.06 (0.87 to 1.30)	
	Copper	Invasive OC				2	1.02 (0.93 to 1.11)	
		LMP OC					1.11 (0.91 to 1.33)	
	Zinc	Invasive OC				3	0.98 (0.91 to 1.06)	
		LMP OC					0.91 (0.77 to 1.08)	
	Calcium	Invasive OC				7	1.24 (0.78 to 1.98)	
		LMP OC					1.90 (0.80 to 4.48)	
	Magnesium, per 0.1 mmol/L increase	Invasive OC				5	0.14 (0.03 to 0.70)	
		LMP OC					0.11 (0.0 to 22.32)	
	Phosphorus	Invasive OC				4	1.16 (0.88 to 1.53)	
		LMP OC					1.04 (0.72 to 1.50)	
	Selenium	Invasive OC				2	1.00 (0.91 to 1.09)	
		LMP OC					0.90 (0.75 to 1.09)	
	Vitamin A	Invasive OC				2	0.82 (0.44 to 1.56)	
		LMP OC					0.56 (0.17 to 2.51)	
	Predicted β-carotene per 0.3 μmol/liter increase	Invasive OC				4	1.04 (1.00 to 1.09)	
		LMP OC					0.82 (0.76 to 0.90)	
	Vitamin B6	Invasive OC				1	1.00 (0.98 to 1.02)	
		LMP OC					1.00 (0.96 to 1.04)	
	Vitamin B12, per 153 pmol/L increase	Invasive OC				15	0.99 (0.92 to 1.06)	
		LMP OC					1.42 (1.21 to 1.68)	
	Vitamin E, per 6 mg/L increase	Invasive OC				3	0.84 (0.47 to 1.52)	
		LMP OC					0.21 (0.06 to 0.76)	
	Folate	Invasive OC				3	0.94 (0.77 to 1.16)	
		LMP OC					0.86 (0.56 to 1.33)	
Larsson et al. (38), 2020	Smoking	Overall OC	25509	40941	OCAC	75	1.06 (0.85 to 1.32)	IVW
			1520	197318	UK Biobank		1.04 (0.95 to 1.14)	
	Alcohol	Overall OC	25509	40941	OCAC	19	1.23 (0.62 to 2.47)	
			1520	197318	UK Biobank		0.91 (0.73 to 1.15)	
Larsson et al, (39), 2020	Insulin-like growth factor-1	Overall OC	25509	40941	OCAC	416	0.95 (0.88 to 1.02)	IVW
			1520	197318	UK Biobank		0.92 (0.78 to 1.08)	
Zhu et al. (40), 2020	Alcohol, drinks per week	Overall OC	25509	40941	OCAC	99	0.83 (0.63 to 1.10),	IVW
	Alcohol use disorder					9	0.92 (0.83 to 1.01)	IVW/Likelihood
	Age-adjusted alcohol use disorder identification					13	0.83 (0.71 to 0.97)	IVW/Likelihood
Ruth et al. (41), 2020	Testosterone	Overall OC	25509	40941	OCAC	254	0.90 (0.83 to 0.97)	IVW
	Sex hormone-binding globulin					359	1.00 (0.89 to 1.13)	
Dimou et al. (42), 2020	Adiponectin	Overall OC	25509	40941	OCAC	18	1.07 (0.96 to 1.19);	IVW
	Leptin					2	1.78 (0.93 to 3.38);	
	sOB-R					4	1.01 (0.99 to 1.03)	
	PAI-1					4	1.22 (0.95 to 1.55);	
Lin et al. (43), 2020	Circulating zinc	Overall OC	25509	40941	OCAC	21	0.97 (0.94 to 1.00)	IVW
		HGSC					0.96 (0.93 to 1.00)	
	Circulating coper	Overall OC				25	1.01 (0.99 to 1.04)	
Larsson et al. (44), 2020	Plasma phospholipid arachidonic acid	Overall OC	25509	40941	OCAC	5	0.97 (0.94 to 1.00)	IVW/WTR
			1520	197318	UK Biobank		1.02 (0.93 to 1.11)	
			311	76077	FinnGen		1.01 (0.84 to 1.22)	
			720	89731	BBJ		0.98 (0.86 to 1.11)	
Yuan et al. (45), 2020	Type 2 diabetes	Overall OC	1520	312191	UK Biobank	399	1.05 (0.97 to 1.12)	IVW
Ye et al. (46), 2021	Vitamin D	Overall OC	552	326096	UK Biobank	104	0.96 (0.93 to 0.99)	IVW
Ong et al. (47), 2021	25-hydroxyvitamin D (per 1 SD increase in 25(OH)D)	Overall OC	25509	40941	OCAC	76	0.89 (0.82 to 0.96)	MR-PRESSO
Ong et al. (48), 2021	Alcohol consumption	Overall OC	22406	40941	OCAC	34	0.95 (0.85 to 1.06)	IVW

BMI, body mass index; BBJ, BioBank Japan; CIMBA, Consortium of Investigators of Modifiers of BRCA1/2; CRP, C-reactive protein; FOCI, Follow-up of Ovarian Cancer Genetic Association and Interaction Studies of the Ovarian Cancer Association Consortium; HGSC, high-grade serous ovarian cancers; IEOC, Invasive epithelial ovarian cancer; LGSC, low-grade serious carcinomas; LMP, Low malignant potential tumours; OCAC, Ovarian Cancer Association Consortium; PCOS, polycystic ovary syndrome; PAI-1, plasminogen activator inhibitor-1; SNP, single nucleotide polymorphism; MR-PRESSO, MR-Pleiotropy Residual Sum and Outlier; MR-RAPs, MR-robust adjusted profile score; IVW; Inverse-variance weighted; WTR, Wald-type ratio.

### Causality Between Life Habits and OC Risk

#### Alcohol Consumption

Alcohol is hypothesized to promote ovarian carcinogenesis based on its potential to increase circulating levels of estrogen and other hormones through its oxidation by-product acetaldehyde, which may act as a co-carcinogen, induction of cytochrome P450 enzymes involved in activation of liver carcinogens, and depletion of folate (49). In contrast, alcohol is reported to prevent ovarian carcinogenesis by decreasing follicle-stimulating hormone levels (50). Observational studies do not support association of alcohol intake with increased risk of OC (51–53). Interestingly, in a subgroup analysis on multiple subpopulations, low alcohol intake was associated with reduced risk of OC while high alcohol intake had the opposite effect (54). Limited reports to date have focused on the causal associations between alcohol and OC risk.

Alcohol is degraded to acetaldehyde in the liver by alcohol dehydrogenase (ADH1) and then to acetate by acetaldehyde dehydrogenase (ALDH2). Carriers of the A-allele of ADH1B rs1229984 consumed less alcohol per week (48). Therefore, early MR studies often use rs1229984 as an instrumental variable. A two-sample MR study based on participants of European ancestry, single instrument MR using rs1229984 and multiple instrument MR using 34 SNPs on alcohol consumption and epithelial OC showed no causal evidence of association (48). In the other two MR studies, similar results were obtained after eliminating interference of potential confounding factors such as body mass index (BMI), smoking, and education (39, 40).

#### Cigarette Smoking

A number of epidemiological studies on epithelial OC have shown that smoking increases risk of OC, but only for the mucinous subtype. Significantly increased risk of invasive mucinous and borderline mucinous OC among current smokers has been reported (55), shown to increase with increased duration of smoking and decline with time after smoking cessation (56). In other studies, smoking was not associated with risk of serous OC and current smokers had a 20% lower risk of developing endometrioid and clear cell OC (57, 58).

An MR study using 115 SNPs from participants of European ancestry recruited from 14 countries reported that lifetime smoking exposure was associated with increased risk of invasive epithelial OC. In subtype-specific analyses, evidence for association of smoking with high grade serous cancer (HGSC), but not the mucinous subtype, was obtained (29). Another MR study on smoking and OC risk with subjects of European descent reported no causal evidence (39).

#### Coffee

Coffee consumption is suggested to be associated with decreased estrogen circulation in pre- and postmenopausal women. Its intake is linked with obesity, metabolic syndrome, and type 2 diabetes as well as liver fibrosis, cirrhosis, and specific types of cancer, including breast, colorectal, lung, endometrial, and prostate cancer. Given that elevated estrogen has been long suspected to increase the risk for OC, coffee consumption may decrease this risk (59). Additionally, risk could be lower because coffee contains flavonoids, and both flavonoids and caffeine have anti-carcinogenic properties. Previous observational studies have shown that coffee intake is potentially associated with reduction of cancer risk. However, prospective studies on the relationship between intake of caffeine and different types of coffee and OC risk have reported conflicting results (60). MR research could aid in clarifying whether this association is causal.

In 2018, Ong et al. (26) conducted MR analysis of moderate coffee consumption and OC risk among subjects of European ancestry. Their results showed no evidence of a strong association between EOC risk and genetically predicted coffee or caffeine levels. In 2019, Ong and co-workers performed a large-scale MR study in a Caucasian British population, with the aim of understanding the causal link between coffee consumption and various cancer types. After several experiments, corrections and meta-analysis, the results of MR remained unchanged. The authors propose that the relationship between coffee intake and disease outcome may have changed due to smoking behavior (33).

### Causality Between Anthropometric Characteristics and OC Risk

Previous studies suggest that anthropometric characteristics are related to OC risk and prognosis (55). While several studies have focused on the role of anthropometric characteristics in risk of OC, the findings to date are inconsistent (55).

#### BMI

Observational studies have revealed an association between BMI and various cancer types. In 2014, fat index was identified as a potential risk factor for OC by World Cancer Research Fund/American Institute for Cancer Research (61). Conversely, according to the US National Cancer Institute, OC is not considered an obesity-related disease. Similarly, the American Cancer Society lists OC as only possibly being linked to overweight or obesity (62). Overall findings from substantial research on adiposity (primarily adult BMI) suggest only a weak positive association, with stronger correlations observed for population-based case–control studies compared to prospective studies. Relatively few studies have conducted detailed examinations of other adiposity-related factors, such as childhood BMI, birth weight, and waist–hip ratio (WHR) (63). The mechanisms by which obesity leads to OC risk remain poorly understood, and the issue of whether associations between obesity and cancer in observational studies are causal is currently unclear.

An MR study published in 2016 with data (all European ancestry) from FOCI and large-scale GWAS of adiposity-related traits comprehensively analyzed the causal relationship between adiposity at different life stages and OC risk. The group reported potential associations of genetic scores for higher adult BMI with increased risk of overall OC but failed to show strong evidence of associations between genetically predicted birth weight, childhood BMI or WHR, and OC risk (21). In 2016, an MR study on the BMI of European adults in relation to risk of different subtypes of OC was published showing that higher genetically predicted BMI was associated with increased risk of non-HGSC but not HGSC cases (22). Secondary analyses stratified by behavior/subtype suggested that consistent with observational data, the strongest association was observed for low-grade/borderline serous OC. Consistent with findings in the general population, MR analysis of height and BMI as modifiers of OC risk in *BRCA1* and *BRCA2* mutation carriers revealed a positive association between BMI and OC risk in premenopausal *BRCA1/2* mutation carriers (32). Subsequent MR analysis showed strong evidence of an association of BMI with invasive epithelial OC. Furthermore, association of BMI with HGSC, endometrioid carcinoma, and low malignant potential tumors but not other subtypes was observed. However, MR-Egger analysis showed little evidence of horizontal pleiotropy (29).

#### Height

Changes in sex hormones in females during their 20s and 30s are important in the pathogenesis of epithelial OC. Height is strongly influenced by the peripubertal hormonal milieu and reflects pubertal hormonal levels. Observational studies support an association of increased height in adults with higher risk of OC (64). Reports of the 2014 World Cancer Research Project Fund/American Institute for Cancer Research have documented convincing evidence of a correlation between adult height and increased OC risk (55). However, these conventional observational studies are subject to inherent bias, including selection bias, differential and non-differential reporting bias, and confounding.

In contrast, an earlier MR study demonstrated little evidence that height is associated with risk of aggressive epithelial OC. In analyses examining histotypes and low malignant potential tumors, significant association of height with clear cell carcinoma was observed, which was robust in various sensitivity analyses, but not with other subtypes (29). In 2018, Dixon-Suen et al. published an MR study on height and OC risk based on data from 16,395 European women with primary ovarian/fallopian tube/peritoneal cancer and 23,003 controls from 39 OCAC studies. The group concluded that greater genetically predicted height was associated with increased OC risk, both overall and separately for invasive and borderline tumors. Among *BRCA1/2* mutation carriers, no causal relationship between height and OC risk was observed (28).

### Causality Between Reproductive Factors and OC Risk

Numerous studies have been performed to establish whether reproductive factors are associated with risk of OC as a gynecological tumor. Infertility has been consistently identified as a risk factor for OC and the use of oral contraceptives, parity, and tubal ligation shown to reduce the risk of disease. In addition, risk of OC is related to use of a number of hormone drugs. Taking into account the effects of pregnancy and use of oral contraceptives on risk of OC, it is reasonable to assume that age at menarche and natural menopause are potential risk factors (65, 66).

#### Age at Menarche

The “incessant ovulation” hypothesis suggests that delaying the age of menarche may reduce the number of ovulations, thereby reducing risk of OC. Moreover, levels of sex hormones (such as progesterone and androgens) show changes during childhood and adolescence, which are thought to play an important role in the etiology of OC. In 2013, a meta-analysis including 22 case–control and 5 cohort studies on age at menarche and OC risk supporting an inverse relationship between menarche and risk of OC was published. An inverse association between menarche age and OC risk has been reported in the majority of subgroups, but limited to invasive and borderline serous OC (65, 67).

Another article showed evidence for association of earlier age at menarche with risk of invasive epithelial OC in inverse-variance-weighted (IVW) models. However, horizontal pleiotropy may bias the IVW estimate. In studies examining invasive epithelial OC histotypes and low malignant potential tumors, evidence for association of earlier age at menarche with endometrioid carcinoma was obtained, which was robust in MR-Egger, weighted median, weighted mode, and leave-one-out analyses (29). MR analysis of women of European descent revealed a strong reverse genetic correlation between age at menarche and BMI. Meanwhile, increasing age at menarche adjusted for genetically predicted BMI was associated with lower risk for OC, in particular, serous OC and endometrial cancer (24). Further MR analysis of Chinese genome-wide association studies and women of European descent revealed a causal relationship between earlier age at menarche and epithelial OC in both Chinese and European populations (34).

#### Age at Natural Menopause

Menopause is permanent cessation of the menstrual cycle, marking the end of female reproductive life. In addition to changes in related sex hormone levels, the timing of menopause can also be applied to predict future health outcomes, such as risk of hormone-related cancers. Earlier menopause may be related to increased risk of OC. This theory is based on the gonadotropin hypothesis for pathogenesis of OC, which predicts that ovarian aging, accompanied by higher concentrations of follicle-stimulating and luteinizing hormones, increases the risk of OC (68). Previous MR analysis of individuals of European descent showed little evidence that late natural menopause is associated with risk of aggressive epithelial OC. However, in subtype-specific analysis, evidence of a potential association of later age of natural menopause with risk of endometrioid carcinoma was obtained (29).

#### Parity

Past epidemiological studies have shown that parity is associated with the occurrence of ovarian cancer. Nulliparity and low parity are associated with an increased risk of ovarian cancer. Parous women have a 30%–40% lower risk of developing ovarian cancer, and an additional protective effect is seen with increasing parity (58). Studies have shown that after the first pregnancy, the risk of ovarian cancer is related to the number of pregnancies, and every pregnancy is related to a reduced risk of ovarian cancer (69). Conversely, MR studies show that there is no relationship between parity and ovarian cancer risk (29).

### Causality Between Pathological Conditions and OC Risk

#### Endometriosis

Endometriosis is a chronic, estrogen-dependent progressive disease characterized by the presence of endometrioid tissue, glands, and interstitium outside the uterine cavity. In addition to serious adverse effects on female health and wellbeing, increased risk of OC development cannot be overlooked. Endometriosis, in particular, ovarian endometriosis, is suggested to increase the risk of malignant tumors. Two main pathways have been proposed to describe the potential association between OC and endometriosis: (1) the two diseases coexist and are the result of common risk factors and their effects and (2) endometriotic cells gradually transform into cancer cells (70). Numerous epidemiological studies have reported a significant increase in incidence of OC in patients with endometriosis. Subsequent retrospective studies consistently demonstrated higher incidence of endometriosis in patients with OC (58). A literature review summarized these findings and indicated that high risk of cancer development was attributable to elevated estrogen concentrations leading to cystic malignant hyperplasia and/or ARID1A gene (SWI/SNF family member) mutations and, consequently, loss of BAF250a expression. Therefore, further exploration of the relationship between endometriosis and OC from a genetic perspective is necessary (70).

Our MR analysis include reports that endometriosis is associated with risk of OC. Strong evidence of an association of genetic liability to endometriosis with increased risk of invasive epithelial OC was obtained in these studies. Subtype-specific analyses further confirmed significant association with clear-cell carcinoma and potential association with endometrioid carcinoma, low malignant potential tumors and HGSC. Findings on invasive epithelial OC and clear-cell carcinoma were reported based on sensitivity analyses examining horizontal pleiotropy whereas somewhat inconsistent effect estimates were found for endometrioid carcinoma, low malignant potential tumors, and HGSC. Analyses employing Steiger filtering provided strong evidence that the causal direction was from genetic liability to endometriosis to invasive epithelial OC whereas the causal direction could not be clearly established for clear-cell carcinoma (29).

#### Polycystic Ovary Syndrome

Polycystic ovary syndrome (PCOS) is a common hormonal disorder affecting 5%–8% women of reproductive age. A population-based case–control study highlighted the possibility of risk of OC in women with PCOS, which was not supported by other studies (71). Recently, the Ovarian Cancer Association Consortium (OCAC) reported decreased risk of invasive OC among women with self-reported PCOS (71, 72). The conflicting results obtained to date highlight the necessity for further research.

Two recent MR analyses on PCOS and OC risk may contribute to clarification of this issue. The first article provided little evidence that genetic susceptibility to PCOS affects the risk of invasive epithelial OC (58). Further subtype-specific analyses revealed an inverse association of genetic liability to PCOS with endometrioid carcinoma, which remained robust in sensitivity analyses. In contrast, association of PCOS with low-grade serous carcinoma was indicated but not clearly detected across all sensitivity analyses in IVW models, suggesting the presence of horizontal pleiotropy or potentially reflecting limited statistical power in these analyses (29). The second study used 14 SNPs to analyze PCOS and risk of OC in women of European descent and demonstrated an inverse association between genetically predicted PCOS and risk of invasive OC. Subtype-specific analyses disclosed the strongest inverse association between genetically predicted PCOS and endometrioid tumors (30).

#### Schizophrenia

For more than 100 years, the debate on whether schizophrenia can reduce the risk of cancer has continued. A number of previous studies indicate that schizophrenia contributes to prevention of cancer. Genetic research additionally supports an inverse correlation between schizophrenia and cancer, including evidence of common protein transcription pathways of the two diseases (73). However, epidemiological studies have not validated this correlation, because no significant differences in cancer risk of patients with varying levels of schizophrenia have been identified (74, 75). A number of researchers suggest that the reduction in cancer risk is attributable to protective genetic effects of schizophrenia while others believe that reduced risk is related to the drugs used to treat schizophrenia (73). From this viewpoint, it is necessary to study schizophrenia in relation to risk of cancer from a genetic perspective.

Choline metabolism disorders in association with schizophrenia and epithelial OC are documented. A bidirectional MR analysis of epithelial OC (data from six OCAC and two Consortium of CIMBA projects) and schizophrenia [Schizophrenia Working Group of the Psychiatric Genomics (76)] highlighted an association of schizophrenia with weaker but increased risk of epithelial OC. Moreover, in subtype-specific analyses, schizophrenia was shown to be associated with increased risk of high-grade serous OC (31).

#### Vitiligo

Vitiligo is an autoimmune disease characterized by selective destruction of melanocytes leading to depigmentation of skin. The association between vitiligo and skin cancer has been discussed previously, but findings to date are inconsistent. The potential correlation between vitiligo and risk of other cancer types has received limited research attention. A recently published MR analysis of vitiligo and cancer risk in European populations suggests a protective role of vitiligo against development of OC (35).

#### Type 2 Diabetes

Several epidemiological studies support an association between type 2 diabetes and increased risk of some types of gynecologic neoplasms, such as endometrial, cervical, ovarian, and vulvar cancer. Insulin resistance, chronic inflammation, and high levels of free ovarian steroid hormones may be among the potential mechanisms underlying this complex relationship (77). In the MR analyses included, there were two studies that mentioned type 2 diabetes and OC risk and showed no evidence of a causal relationship (29, 45).

### Causality Between Nutritional Factors and OC Risk

Nutritional factors are related to OC, and improper lifestyle choices can exacerbate disease progression. Therefore, assessment of the impact of diet on risk of OC is of critical importance to the public, clinicians, and research and health institutions (78). MR research on nutritional factors and OC risk could provide a fundamental understanding of this association.

#### Vitamin A

Vitamin A activity is important for normal control of cellular differentiation and proliferation and hypothesized to modify cancer risk. Interestingly, a previous study exploring the correlation between vitamin A levels and risk of OC demonstrated no association while a subsequent meta-analysis reached the opposite conclusion (79, 80). A further MR analysis using two SNPs on a Caucasian population showed no causal link between vitamin A levels and risk of OC (37).

#### Vitamin E

Vitamin E, also designated tocopherol, has strong antioxidant activity that protects cells against oxidative DNA damage and mutagenesis, thereby preventing the onset of specific tumors. Vitamin E also contains putative anti-cancer and anti-mutant compounds and were suggested to play a role in the prevention of cancer. However, conflicting data have been reported showing that vitamin E is not related to OC (80, 81). An MR analysis focusing on three SNPs in a European population showed that this study showed no association between vitamin E and OC risk. However, in a study conducted on invasive epithelial OC and low malignant potential cancers, genetically predicted vitamin E levels were inversely associated with these cancer types (37).

#### B Vitamins

B vitamins (including folate, thiamin, riboflavin, niacin, vitamin B6, and vitamin B12) are essential micronutrients purported to influence carcinogenesis through regulation of one-carbon metabolism. Women in the highest quintile of folate and vitamin 6 intake were shown to have lower risk of OC than those in the lowest quintile (80, 82). However, an MR analysis of European populations showed that higher vitamin B12 concentration was associated with increased risk of low malignant ovarian tumors while other B vitamins (B6, folate) are not associated with risk of OC (37).

#### Vitamin D

Vitamin D has attracted widespread scientific interest in cancer prevention research. Data from *in vitro* and animal model studies support anti-tumor effects of vitamin D (83). Vitamin D functions by activating the nuclear vitamin D receptor, which is ubiquitously expressed and regulates the growth, differentiation, and apoptosis of normal and tumor cells. However, evidence from case–control and cohort studies so far suggests no effect of vitamin D on OC risk and survival (84).

The results of the three earlier MR studies may provide further insights into the potential association of vitamin D with OC. In 2016, Ong et al. failed to find a link between vitamin D and the risk of ovarian cancer (20). In 2017, Dimitrakopoulou et al. conducted an MR study of vitamin D using four SNPs to evaluate multiple cancer risk in women of European ancestry. The group failed to show a causal relationship between circulating vitamin D concentrations and OC risk (23). Similarly, a study published by Ong et al. in 2018 still failed to find a link between vitamin D and the risk of ovarian cancer (27). In 2021, an MR study by Ye et al. (46) using 104 SNPs on women of European descent showed that higher circulating vitamin D concentrations can reduce the risk of OC. The latest study by Ong and co-workers in the face of horizontal pleiotropy involving analysis with 74 SNPs further validated this result. Increase in vitamin D concentration may thus be related to decreased risk of OC (47).

#### β-Carotene

As a main vitamin precursor, vitamin A carotenoid, β-carotene is metabolized into biologically active retinol and other vitamin A compounds essential for maintenance of normal human physiology and homeostasis. Previous *in vitro* and *in vivo* studies have shown that β-carotene is a powerful antioxidant that can neutralize free radicals in cells involved in the development of chronic diseases. However, similar to other active substances with antioxidant properties, variable results have been obtained on potential associations of β-carotene and various cancers (85, 86).

Data from MR studies on β-carotene and OC may provide evidence for related research. In standard IVW analysis, genetically predicted serum β-carotene levels were positively associated with invasive epithelial OC, mucinous carcinoma, and endometrioid carcinoma. Conversely, β-carotene levels were negatively correlated with low-grade serous carcinoma, low malignant potential tumors, and mucinous borderline tumors (37).

#### Selenium

Selenium is an important trace element in the human body. A lack of trace elements necessary to maintain balance in the body, such as cofactors, and accumulation of specific toxic metals, may destroy resistance of the host to cancer. For example, selenium is a critical component of selenoproteins and plays a key role in resistance to oxidative stress. A number of epidemiological studies support an inverse correlation of selenium levels with cancer, in particular, breast cancer (87). Similar to the results of other epidemiological investigations, no causal relationship between selenium and OC was observed in the MR study (37).

#### Phosphorus

Phosphorus is one of the main elements that widely affect health of organisms. Almost all natural foods contain phosphorus in the form of inorganic phosphate or organic molecules. Several tumor types are reported to be associated with high phosphorus intake, including lung, colon, breast, ovary, and endometrial cancer, among others (88). In contrast, no causal link between circulating phosphorus concentrations and risk of OC was detected in an MR study (37).

#### Metal Elements

Iron is an essential element for numerous cellular processes. Imbalance in homeostasis attributable to iron overload is harmful to the body (89) and believed to contribute to the onset of cancer. Considering the known functions of oxidative stress, DNA repair, cell cycle regulation, and angiogenesis, trace metal concentrations in the diet (including zinc and copper) can affect cancer risk. Previous studies clearly suggest that circulating zinc and copper status are associated with initiation of OC (90, 91). Increasing evidence supports the synergistic roles of calcium and vitamin D in physiological processes. A recent randomized clinical trial reported that calcium supplementation reduces the risk of all-cause cancer in women and simultaneous supplementation with calcium and vitamin D exerts greater protective effects (92, 93). In addition, accumulating literature indicates that the balance between calcium and magnesium intake (Ca:Mg ratio) may modify the relationship between calcium and magnesium intake and risk of various outcomes (94).

Although various avenues of research on metal elements in relation to OC are ongoing, MR analysis remains an important means to clarify causal relationships. The MR study specified above highlighted an association of increase in magnesium concentration with decreased risk of epithelial OC. However, no causal relationship has been uncovered between other metal ions and risk of OC (37). Notably, a recently published MR study using 21 SNPs as instrumental variables on circulating copper and zinc and risk of OC in subjects of European ancestry showed novel results distinct from previous findings. Their data suggest that the circulating zinc concentration is causally related to risk of OC, in particular, HGSC (43).

### Causality Between Biomarkers and OC Risk

#### C-Reactive Protein

C-reactive protein (CRP) is a highly sensitive and widely used systemic marker of inflammation. The protein is mainly produced by liver cells, together with other acute phase proteins, and released into the circulatory system in response to tissue damage and inflammation. Systematic reviews and meta-analyses have validated the utility of serum CRP levels as an effective indicator of risk of OC (95). However, further research is essential to clarify the causal relationship between CRP and risk of OC and the role of CRP in etiology of disease.

MR analysis conducted on a European population showed that despite no evidence that C-reactive protein affects risk of invasive epithelial OC, analyses examining histotypes and low malignant potential tumors suggested an inverse association of C-reactive protein with endometrioid carcinoma. C-reactive protein was not clearly associated with other histotypes or low malignant potential tumors (29).

#### Sex Hormone Binding Globulin

The sex hormone binding globulin (SHBG) gene regulates its effect by regulating the bioavailability of sex steroid hormones in target tissues (such as ovary). Hormone stimulation of ovarian epithelial cells is proposed as a mechanism underlying the development of OC. According to animal and *in vitro* studies as well as epidemiological observations, available evidence that sex steroids play a role in OC is mainly indirect and the precise relationship between circulating levels of sex steroids and risk of OC is yet to be established (96). An earlier MR analysis of a population of European descent showed little evidence of an association of genetic liability to sex hormone binding globulin with OC or its subtypes (29). In 2020, an MR analysis of testosterone and cancer showed the same results (41).

#### HMG-CoA Reductase

Statins are widely used to treat hypercholesterolemia. These drugs inhibit 3-hydroxy-3-methyl-glutaryl-CoA reductase (HMGCR), an enzyme necessary for synthesis of mevalonate (97). HMGCR is essential for cellular synthesis of cholesterol and various non-steroidal isoprenoid derivatives involved in proliferation, differentiation, and survival (98). Both *in vitro* and *in vivo* studies have shown that statins inhibit cancer cell growth by inducing apoptosis and inhibiting cell cycle progression through multiple cell signaling pathways (99). MR studies could be effectively used to explore the causal relationship between HMG-CoA reductase inhibition and risk of OC. An MR study in which all participants were of European descent (median age of the cohort, 41.5 to 59.0 years) showed that genetically proxied HMG-CoA reductase inhibition equivalent to 1 mmol/L (38.7 mg/dl) reduction in LDL cholesterol is associated with lower odds of epithelial OC. Similarly, in BRCA1/2 mutation carriers, genetically proxied HMG-CoA reductase inhibition was associated with lower OC risk (36).

#### Insulin-Like Growth Factor 1

Due to the increase in cardiovascular, endocrine, and metabolic diseases, such as metabolic syndrome, diabetes and polycystic ovary syndrome, the prevalence of insulin resistance continues to increase. Several studies support a link between insulin resistance and OC. Insulin resistance is reported to be related to ovarian steroid hormone imbalance and inflammation in diabetic patients and gynecological malignancies. Effective control of insulin resistance could therefore prevent various gynecological cancers. However, contrary to these findings, no association between insulin-like growth factor 1 (IGF-1) or binding protein 3 (IGFBP-3) and OC was identified in other studies (100). The causal link between IGF-1 and the risk of OC is also an issue of concern. A previous MR study on insulin-like growth factor-1 and site-specific cancer risk in a population of European descent demonstrated no significant association between genetically predicted IGF-1 levels and 14 other cancers (including OC), with the exception of colorectal cancer (44).

#### Testosterone

Testosterone is also a key hormone in women. In addition to being an essential precursor for estradiol biosynthesis, testosterone directly acts as an androgen and exerts physiological effects on both reproductive and nonreproductive tissues in women. The role of endogenous androgens in ovarian carcinogenesis is not well understood at present. A number of reports have shown no correlation between androgens and overall risk of invasive epithelial OC while other studies suggest that androgens are both protective and carcinogenic (101, 102). MR research conducted from a genetic perspective may provide constructive perspectives on the relationship between testosterone and OC risk. An MR study on the impact of testosterone on diseases in both sexes highlighted that genetically higher levels of testosterone are harmful to women with metabolic diseases and increase the risk of endometrial cancer but reduce risk and of OC (41).

#### Arachidonic Acid

Arachidonic acid (AA) is metabolized by cyclooxygenases and lipoxygenases to proinflammatory eicosanoids that modulate tumor cell proliferation, differentiation, and apoptosis according to experimental research. AA is a polyunsaturated fatty acid present at high concentrations in the OC microenvironment and associated with poor clinical outcomes. Several studies support its utility as a therapeutic target for intervention and prognostic indicator of OC (103). However, an MR study on female patients of European descent revealed no association of AA with OC risk (38).

#### Circulating Adipokine Concentrations

Obesity is considered a chronic inflammatory state characterized by continued infiltration of adipose tissue by macrophages and other immune cells leading to increased or decreased adipose secretion of adipokines [such as adiponectin, leptin, and plasminogen activator inhibitor-1 (PAI-1)] that may be linked to cancer development (104, 105). While accumulating research suggests that obesity presents an important risk factor for development of OC (61, 62), the underlying molecular mechanisms are not fully understood. Obesity is proposed to lead to increased insulin signaling, inflammation, enhanced availability of lipids, and changes in adipokine signaling, resulting in transformation of normal epithelial cells into aggressive tumor cells (106). Conversely, a previous large-scale MR study on circulating adiponectin and five obesity-related cancer types does not support an association of tumor progression with concentrations of circulating adiponectin, leptin, sOB-R and PAI-1. The causal relationship between circulating adipokines and development of obesity-related cancers (including OC) is yet to be established (42).

#### Telomere Length

Telomeres, which protect the physical integrity of linear chromosomes, are shortened with each cell division, a process that may be accelerated by damage incurred by oxidative stress. Tissue-based studies have revealed a pattern of telomere shortening, genomic instability, and upregulated telomerase expression in many tumor types, including OC. As cells progress from noninvasive precursor lesions to cancer, telomere shortening is a common phenomenon of the early stage of malignant transformation. Prospective studies suggest that greater circulating leukocyte telomere length is associated with lower risk of OC, especially for non-serous and rapid death cases. However, no evidence showing that overall telomere length is causally related to the risk of OC is currently available (107). In 2015, an MR study on telomere length in relation to common cancers in subjects of European descent was published. The study used 11 SNPs as instrumental variables and showed no causal relationship between telomere length and OC and its subtypes (19). However, a more recent MR study published in 2017 using 16 SNPs as instrumental variables on subjects of European descent showed a significant causal relationship of longer telomere length with increased risk of serous low malignant potential OC (25).

## Discussion

MR is effective in reducing reverse causality and confounding variables and has gradually become an increasingly useful tool in epidemiological research. Moreover, MR analysis can be effectively used to analyze exposures that are not easy to investigate in some RCT and observational studies (such as height and BMI) (11, 16). The key to MR analysis is use of SNP as an instrumental variable to explore the relationship between exposure and results. Therefore, even under conditions of exploring the same exposure, when different SNPs or different numbers of SNPs are included, the results of MR analysis may differ, which may explain the variable findings discussed above. According to the classification of risk factors, we have sorted out the MR research and research results related to OC from 2015 to the present in detail. Readers can directly and comprehensively understand the application of MR research in the field of OC by reading this article.

A straightforward and common way of performing MR is called the ratio of coefficients or Wald method. The causal effect is triangulated by dividing the coefficients of regression of the outcome on the IV by the regression of the exposure on the IV (108). This method can be performed using summary-level data, without the need for individual-level data (108). Two-stage least-squares method is another method of performing MR analysis. Two-stage least-squares method involves two stages of regression: The first is from the IVs to the exposure, and the second is from the exposure to the outcome (108). However, this method requires individual-level data and becomes biased when at least one invalid IV is used (109).

Despite the fact that the inverse-variance weighting method gives higher weighting to SNPs, it makes the standard errors in the IV-outcome regression smaller (110). A number of limitations must be considered. A common issue is horizontal pleiotropy, which is difficult to avoid in MR research. Horizontal pleiotropy indicates that instrumental variables are not directly related to results through exposure, which violates the third hypothesis of instrumental variables (111). For the horizontal pleiotropy of one-sample MR, the Q test has a good test effect, especially when the data set is large, but the Q test cannot explain the origin of the horizontal pleiotropy (112). Some of the MR studies we included use the Q test, such as Yarmolinsky et al. (36). Another method that serves as a sensitivity analysis is an adaptation of Egger regression called MR-Egger. It can be used to detect bias that results from horizontal pleiotropy based on the assumption that any pleiotropic effects from IVs on the outcome are independent of the exposure (113). This method is widely used in the studies we included. In addition, in recent years, such as Larsson et al. (44), 2020, MR-PRESSO can minimize and correct the level of pleiotropy, but only if the traits that cause horizontal pleiotropy was known a *priori* (114). On this basis, the weighted median method gives consistent results when at least 50% of the IVs are valid (109) and weighted mode methods can infer a causal effect, even if the majority of IVs are invalid (115).

In addition, the bias in MR can also originate from assortative mating, that is, nonrandom matching between spouses (116). Whether it is a single-trait assortative mating, for example, tall women are more likely to select tall men, or a cross-trait assortative mating (117), for example, women with high intelligence test scores select taller men in research (118), results will be biased due to the non-random nature of this mating. This kind of bias is more common in MR studies where appearance characteristics such as height are used as exposure factors (119). Unfortunately, the two height-related MR studies included in our study did not consider the issue of assortative mating. In these two studies, statistical methods were not used to deal with the bias caused by assortative mating.

Similarly, linkage disequilibrium, defined as a nonrandom association between alleles at a genetic locus on a chromosome, which violates the basic assumption of instrumental variables (6), is a common occurrence. The Bayesian test that can be used to determine whether the association is the result of a colocalized SNP may also reduce the linkage disequilibrium bias in MR analysis (120). As well as setting a maximum pairwise linkage disequilibrium threshold for SNP inclusion, methods such as penalized logistic regression have been described as a means of selecting SNPs based on the knowledge of linkage disequilibrium (121). Some of the MR studies we included use the penalized logistic regression, such as Ong et al. (20). In addition, the winner’s curse is also a situation that has sometimes appeared in past MR studies. In the context of GWAS, the winner’s curse refers to the situation that usually only the main SNP with the smallest *P* value is reported, and other important SNPs may not even be mentioned (122). This makes the statistical ability of MR analysis insufficient. This situation often occurs in one-sample MR analysis due to chance correlation between instrumental and confounding variables during the discovery stage of the GWAS (123). Two-sample MR analysis can solve this problem well. Most of the MR analyses we have included are two-sample MR analyses.

Weak instrument bias occasionally appears in MR research, such as the IVs explain only a small part of the resulting phenotype (124). This then leads to a bias towards the confounded observational association or the null hypothesis, respectively, depending on whether one- or two-sample MR was used (123). Therefore, the *F*-statistic regression of the exposure on the IV is generally used to define strength, defining an instrument as being weak with a score lower than 10 (125). The *I* ^2^ statistic may be used to check for weak instrument bias in MR-Egger analysis; values closer to 0 may be indicative of weak instrument bias (126).

In the context of MR analysis, the collider is a variable, which is the causal downstream of exposure and result (15). When trying to make statistical adjustments or conditioning to the collider, bias may occur (127, 128). This means that sample selection may introduce bias into MR analysis. Selection bias is considered to be a form of collider bias. Inverse probability weighting is a countermeasure to collider/selection bias (127). Inverse probability weighting considers underrepresented cases in the data set and gives them more weight in the analysis, assuming that these cases may be more common in the general population (127).

With the continuous development of GWAS, we should be able to successfully identify further accurate exposure-related SNPs as instrumental variables for continued MR analysis of specific exposures and findings to establish causal relationships. With the enrichment of statistical methods and the deepening of observational research, the results of MR analysis will become more accurate and reliable.

## Conclusion

In conclusion, MR analysis plays an important role in etiological research on OC. Overall, higher BMI and height, earlier age of menarche, endometriosis, schizophrenia, and higher circulatory β-carotene and circulatory zinc levels are associated with increased risk of OC. Conversely, PCOS; vitiligo; higher circulatory vitamin D, magnesium, and testosterone levels; and HMG-CoA reductase inhibition are associated with reduced risk of OC. Despite its limitations, MR analysis should provide constructive insights into disease prevention and drug development as well as effective guidance for observational research and RCT.

## Author Contributions

J-ZG, Q-JW, and T-TG designed the study and formulated the clinical question. J-ZG, QX, and Q-JW performed the literature search and reviewed the search results for study inclusion. J-ZG, QX, and Q-JW designed the data extraction form and extracted the data. All authors collected, managed, and analyzed the data. J-ZG, QX, and Q-JW drafted the manuscript. All authors prepared, reviewed, revised, and approved the manuscript. Q-JW and T-TG had full access to all data in the study and is responsible for data integrity and the accuracy of data analysis. J-ZG and Q-X contributed equally to this work. All authors contributed to the article and approved the submitted version.

## Funding

This study was supported by grants from the Natural Science Foundation of China (No. 82073647 to Q-JW), the LiaoNing Revitalization Talents Program (No. XLYC1907102 to Q-JW), the Shenyang High Level Innovative Talents Support Program (No. RC190484 to Q-JW), and the 345 Talent Program to Q-JW (No. M0268).

## Conflict of Interest

The authors declare that the research was conducted in the absence of any commercial or financial relationships that could be construed as a potential conflict of interest.

## Publisher’s Note

All claims expressed in this article are solely those of the authors and do not necessarily represent those of their affiliated organizations, or those of the publisher, the editors and the reviewers. Any product that may be evaluated in this article, or claim that may be made by its manufacturer, is not guaranteed or endorsed by the publisher.
